# Exploring causal associations between autoimmune diseases and hearing loss: a mendelian randomization study

**DOI:** 10.1016/j.bjorl.2026.101837

**Published:** 2026-05-28

**Authors:** Yutong Huang, Shengli Gao, Renzhong Luo

**Affiliations:** Guangzhou Medical University Women and Children′s Medical Center, Guangdong Provincial Clinical Research Center for Child Health, Department of Otolaryngology Head and Neck Surgery, Guangzhou, Guangdong, China

**Keywords:** Autoimmune diseases, Hearing loss, Sensorineural hearing loss, Mendelian randomization analysis, Genome-wide association study

## Abstract

**Objective:**

The causal relationship between Autoimmune Diseases (ADs) and Hearing Loss (HL) remains unclear. This study investigates whether genetic predispositions associated with ADs contribute to HL risk.

**Methods:**

Mendelian Randomization (MR) analysis was conducted to explore the causal effects of ADs on HL. SNPs from Genome-Wide Association Studies (GWAS) were used as instrumental variables for ADs, including Rheumatoid Arthritis (RA), Type 1 Diabetes (T1D), Systemic Lupus Erythematosus (SLE), Sjögren’s Syndrome (SS), Ankylosing Spondylitis (AS), Multiple Sclerosis (MS), Crohn's Disease (CD), and Ulcerative Colitis (UC). Outcome data included Sensorineural Hearing Loss (SNHL), Conductive Hearing Loss (CHL), Mixed conductive and sensorineural Hearing Loss (MHL), and Sudden Idiopathic Hearing Loss (SIHL). MR analyses employed Inverse Variance Weighted (IVW) as the primary method, supplemented with MR-Egger, weighted median, and weighted mode. Heterogeneity, pleiotropy, and sensitivity were evaluated using Cochran's *Q* test, MR-Egger regression, MR-PRESSO, and leave-one-out analysis.

**Results:**

The IVW method identified nine significant associations: MS-SIHL (OR = 1.0494, 95% CI 1.0072–1.0934), AS-CHL (OR = 1.2832, 95% CI 1.0643–1.5472), AS-MHL (OR = 1.5994, 95% CI 1.3696–1.8678), AS-SNHL (OR = 1.1903, 95% CI 1.1104–1.276), AS-SIHL (OR = 1.481, 95% CI 1.22–1.798), SLE-CHL (OR = 1.0593, 95% CI 1.0116–1.1092), UC-MHL (OR = 1.0907, 95% CI 1.0027–1.1865), CD-CHL (OR = 1.0529, 95%CI: 1.0074–1.1005), and CD-SIHL (OR = 1.0597, 95% CI 1.0177–1.1034). Among these, outliers were detected only in AS-SNHL. After outlier removal, the AS-SNHL association remained significant (OR = 1.1722, p < 0.00001), with resolved heterogeneity and pleiotropy. No heterogeneity and pleiotropy were found for the other associations.

**Conclusion:**

This study identified nine significant AD-HL associations, emphasizing the need for targeted screening and management of HL in individuals with AD.

**Level of Evidence:**

Level 5.

## Highlights

• MR analysis uncovers causal links between autoimmune diseases and hearing loss.
• Ankylosing spondylitis shows significant ties to various hearing loss types.
• Inflammatory bowel diseases are linked to multiple hearing loss subtypes.
• Early audiological assessment is needed for patients with autoimmune diseases.

## Introduction

Hearing Loss (HL) is a common sensory disorder affecting individuals of all ages, with significant impacts on quality of life. It is broadly classified into Sensorineural Hearing Loss (SNHL), Conductive Hearing Loss (CHL), and Mixed conductive and sensorineural Hearing Loss (MHL).^1^ SNHL, caused by damage to the cochlea or auditory nerve, is the most common type, with sudden cases (Sudden Idiopathic Hearing Loss; SIHL) affecting 5–27 per 100,000 annually in the United States and prevalence increasing significantly with age.^2^ CHL, caused by impaired sound transmission in the outer or middle ear, commonly arises from otitis media and affects approximately 1.7% of children globally, with a substantially higher burden in low- and low-middle-income regions.^3^ MHL combines elements of both. Risk factors for HL include genetic predisposition, infections, ototoxic medications, noise exposure, and systemic conditions such as Autoimmune Diseases (ADs).^4^^,^^5^

ADs are conditions characterized by immune system dysregulation, leading to attacks on the body’s own tissues, with examples including Rheumatoid Arthritis (RA), Type 1 Diabetes (T1D), Systemic Lupus Erythematosus (SLE), and Sjögren’s Syndrome (SS).^6^ These diseases share mechanisms like autoantibody production, chronic inflammation, and cytokine imbalances, resulting in tissue damage and impaired immune tolerance.^7^ ADs are increasingly recognized as risk factors for HL due to their potential to mediate immune-driven damage to auditory structures.^8^ Mechanistically, autoantibodies and inflammatory cytokines disrupt the blood-labyrinth barrier, impair cochlear microcirculation, and induce oxidative stress, leading to hair cell apoptosis and auditory dysfunction.^9^ Chronic inflammation further destabilizes neural signaling and endolymphatic homeostasis, exacerbating HL.^10^ These processes highlight the vulnerability of auditory structures to immune-mediated damage.

While the association between ADs and HL has been reported, the causal relationship remains unclear. To address this, Mendelian Randomization (MR) analysis offers a robust approach to investigate potential causality between ADs and HL. MR utilizes genetic variants, typically Single Nucleotide Polymorphisms (SNPs) identified through Genome-Wide Association Studies (GWAS), as Instrumental Variables (IVs) to assess the causal relationship between an exposure and an outcome.^11^ By minimizing confounding and reverse causality, MR provides a reliable method to identify causal links that may not be feasible to explore through randomized controlled trials.^12^ This study employs a two-sample MR approach to investigate the genetically predicted causal relationship between ADs and HL. The findings may contribute to a better understanding of their connection and inform future research on preventive and therapeutic approaches.

## Methods

### Study design

**Fig. 1:**
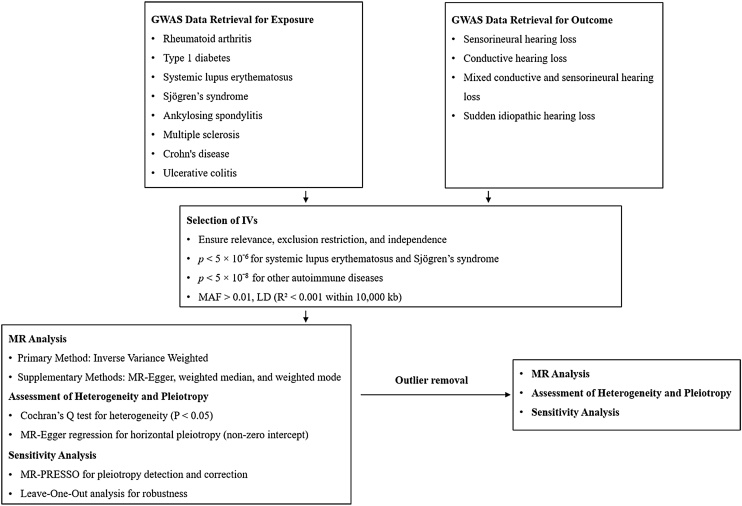
Study flowchart for Mendelian Randomization (MR) analysis of Autoimmune Diseases (ADs) and Hearing Loss (HL). The flowchart outlines the methodology for investigating causal relationships between ADs and various types of HL using MR. Genome-Wide Association Study (GWAS) data retrieval included datasets for Rheumatoid Arthritis (RA), Type 1 Diabetes (T1D), Systemic Lupus Erythematosus (SLE), Sjögren's Syndrome (SS), Ankylosing Spondylitis (AS), Multiple Sclerosis (MS), Crohn’s Disease (CD), and Ulcerative Colitis (UC), as well as outcomes for Sensorineural Hearing Loss (SNHL), Conductive Hearing Loss (CHL), Mixed conductive and sensorineural Hearing Loss (MHL), and Sudden Idiopathic Hearing Loss (SIHL). Instrumental Variable (IV) selection ensured relevance, independence, and exclusion restriction, with strict thresholds for Single Nucleotide Polymorphism (SNP) significance. MR analysis primarily used the Inverse Variance Weighted (IVW) method, supplemented by MR-Egger, weighted median, and weighted mode. Sensitivity analyses included Mendelian Randomization Pleiotropy Residual Sum and Outlier (MR-PRESSO) for pleiotropy correction, leave-one-out analysis for robustness, and heterogeneity tests using Cochran’s *Q* and MR-Egger regression. Outlier removal was followed by a second round of MR analysis, along with reassessment of heterogeneity, pleiotropy, and sensitivity.

The methodology followed the STROBE-MR statement guidelines. Relevant GWAS databases were selected to obtain the appropriate SNPs. IVs were selected based on the three key principles of MR analysis: relevance, independence, and exclusion restriction.^13^ MR analysis was then performed to investigate causal relationships. Sensitivity was assessed using various statistical methods to ensure the robustness of the findings, providing a thorough understanding of the potential associations between ADs and HL risk. The study flowchart is illustrated in Fig. 1.

### Data sources

Exposure data were sourced from the IEU Open GWAS database and included eight common ADs: RA, T1D, SLE, SS, Ankylosing Spondylitis (AS), Multiple Sclerosis (MS), Crohn's Disease (CD), and Ulcerative Colitis (UC) based on a published study.^14^ Outcome data on HL were derived from the FinnGen R11 database (https://www.finngen.fi/en/access_results), including SNHL, CHL, MHL, and SIHL. Both exposure and outcome GWAS were restricted to individuals of European ancestry to minimize population stratification. Outcome data were obtained from Finnish participants, whereas exposure data were not country-specific. All GWAS data used in this study were derived from previously published studies with documented ethical approval and informed consent. Detailed information on these datasets is provided in Table S1.

### IV selection

SNPs significantly associated with SLS and SS were included with a threshold of p <5 × 10^−6^, due to difficulties in identifying sufficient SNPs that meet stricter significance levels. For other ADs, a threshold of p <5 × 10^-8^ was applied.^15^ Only SNPs with a Minor Allele Frequency (MAF) >0.01 were retained.^16^ Linkage Disequilibrium (LD) was minimized by excluding SNPs with R^2^ > 0.001 within a 10,000 kb window.^17^ Proxy SNPs with R^2^ > 0.8 were used if IVs were absent in the outcome data.^18^ F-statistics (F = R^2^ × (N-2) / (1-R^2^)) were calculated to ensure IV strength (F > 10) and prevent weak instrument bias. R² represents the proportion of variance in the exposure explained by the SNP.^19^

### MR analysis

The Inverse Variance Weighted (IVW) random effects method was the primary approach, providing Odds Ratios (ORs) with 95% Confidence Intervals (CIs) by calculating a weighted average effect size based on SNP inverse variance.^20^ To ensure robustness, MR-Egger, weighted median, and weighted mode methods were applied. MR-Egger accounts for pleiotropy by including an intercept term,^21^ the weighted median provides reliable estimates if at least 50% of IVs are valid,^22^ and the weighted mode identifies the causal effect as the precision-weighted mode, even when most IVs are invalid.^23^

### Sensitivity analysis

Sensitivity analyses were performed to ensure the robustness of the findings and detect pleiotropy. Heterogeneity was assessed using the IVW method with Cochran's *Q* test. A p-value >0.05 indicates low heterogeneity, suggesting a minimal influence on IVW results.^24^ Pleiotropy was evaluated using MR-Egger regression and the MR-PRESSO. MR-Egger regression assessed horizontal pleiotropy through its intercept, with values close to zero or non-significant indicating no pleiotropy.^25^ MR-PRESSO identified and corrected horizontal pleiotropy by excluding outlier SNPs.^26^ Leave-one-out analysis was performed by sequentially removing each SNP to evaluate its influence on the overall estimates and assess the stability of the results.^27^

### Software and data visualization

Forest, scatter, and funnel plots were used for visualization. All analyses were performed using *R* version 4.0.5 and the “TwoSampleMR” package.

## Results

### IV selection

The study selected 21 IVs for UC, 36 IVs for T1D, 15 IVs for SLE, 10 IVs for SS, 25 IVs for RA, 22 IVs for MS, 52 IVs for CD, and 26 IVs for AS. The mean *F*-values for these IVs ranged from 30.491 for SLE to 153.719 for AS, indicating strong instrument strength (Table S2). Proxy SNPs were identified for SIHL, SNHL, MHL, and CHL across various exposures. For example, rs2853953 replaced rs1131114 in SLE for all four outcomes (Table S3). Palindromic SNPs were identified for various exposures, including rs12692254 for CD, rs3093017 for RA, and rs4065985 and rs7936434 for UC (Table S4).

### MR analysis

**Fig. 2:**
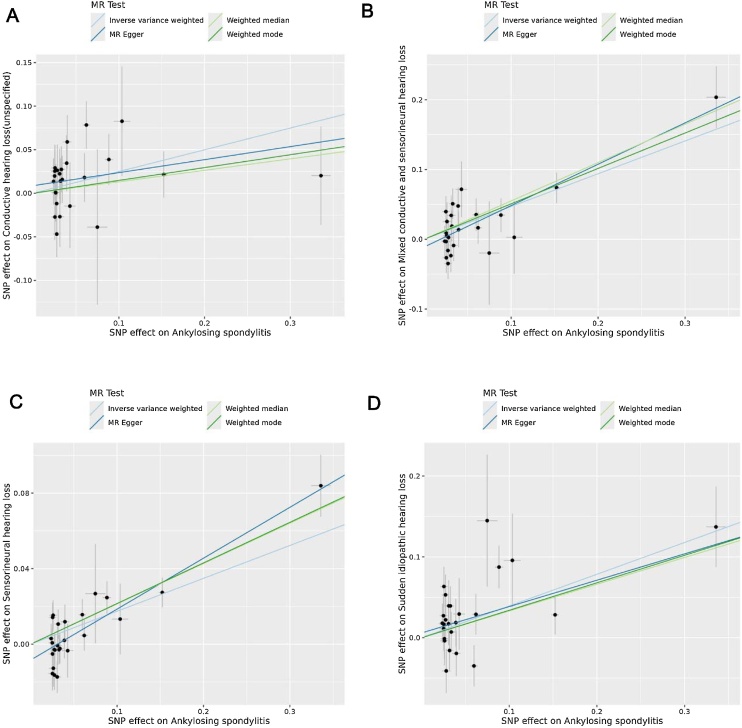
Scatter plots show genetic associations between AS and HL subtypes identified in the primary MR analysis. (A) AS association with CHL (unspecified); (B) AS association with MHL; (C) AS association with SNHL; (D) AS association with SIHL. Regression lines for IVW, MR-Egger, weighted median, and weighted mode methods are included.

**Fig. 3:**
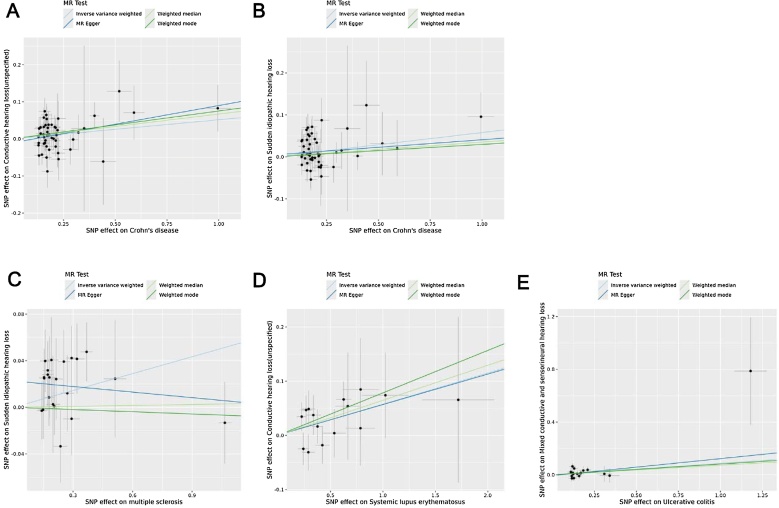
Scatter plots show genetic associations between CD and CHL (A), CD and SIHL (B), MS and SIHL (C), SLE and CHL (D), UC and MHL (E). Regression lines for IVW, MR-Egger, weighted median, and weighted mode methods are included.

MR analysis using the IVW method revealed nine significant AD–HL associations, including MS-SIHL (OR = 1.0494, 95% CI 1.0072–1.0934, p = 0.0213), AS-CHL (OR = 1.2832, 95% CI 1.0643–1.5472, p = 0.009), AS-MHL (OR = 1.5994, 95% CI 1.3696–1.8678, p < 0.00001), AS-SNHL (OR = 1.1903, 95% CI 1.1104–1.2760, p < 0.00001), AS-SIHL (OR = 1.481, 95% CI 1.22–1.798, p = 1.00E-04), SLE-CHL (OR = 1.0593, 95% CI 1.0116–1.1092, p = 0.0142), UC-MHL (OR = 1.0907, 95% CI 1.0027–1.1865, p = 0.0431), CD-CHL (OR = 1.0529, 95% CI 1.0074–1.1005, p = 0.0222), and CD-SIHL (OR = 1.0597, 95% CI 1.0177–1.1034, p = 0.005) (Table 1). The complete results of the MR analysis are shown in Table S5. Scatter plots (Figs. 2‒3 ) and forest plots (Fig. S1‒S2) illustrate these significant associations and SNP-specific effect estimates.

**Table 1:** MR analysis of autoimmune diseases and hearing loss subtypes using the IVW method.

Exposure	Outcome	N.SNPs	Methods	OR (95% CI)	p
Multiple sclerosis	Sudden idiopathic hearing loss	22	IVW	1.0494 (1.0072–1.0934)	0.0213
Ankylosing spondylitis	Conductive hearing loss (unspecified)	25	IVW	1.2832 (1.0643–1.5472)	0.009
Ankylosing spondylitis	Mixed conductive and sensorineural hearing loss	25	IVW	1.5994 (1.3696–1.8678)	<0.00001
Ankylosing spondylitis	Sensorineural hearing loss	25	IVW	1.1903 (1.1104–1.276)	<0.00001
Ankylosing spondylitis	Sudden idiopathic hearing loss	25	IVW	1.481 (1.22–1.798)	1.00E-04
Systemic lupus erythematosus	Conductive hearing loss (unspecified)	15	IVW	1.0593 (1.0116–1.1092)	0.0142
Ulcerative colitis	Mixed conductive and sensorineural hearing loss	18	IVW	1.0907 (1.0027–1.1865)	0.0431
Crohn's disease	Conductive hearing loss (unspecified)	50	IVW	1.0529 (1.0074–1.1005)	0.0222
Crohn's disease	Sudden idiopathic hearing loss	50	IVW	1.0597 (1.0177–1.1034)	0.005

### Heterogeneity and pleiotropy analysis

Significant heterogeneity was observed in ten AD-HL associations, including MS-MHL, MS-SNHL, AS-SNHL, T1D-SNHL, SS-CHL, RA-MHL, RA-SNHL, SLE-MHL, SLE-SNHL, and CD-MHL, with *Q*-statistic ranging from 16.01 to 161.36 (all p < 0.05). MR-Egger regression analysis identified significant horizontal pleiotropy in AS-SNHL association (MR-Egger intercept = −0.00839, p = 0.006593; Table 2). The complete results of the Heterogeneity and pleiotropy analysis analysis are shown in Table S6. No Heterogeneity and pleiotropy were observed in other significant IVW associations. Funnel plots illustrated horizontal pleiotropy (Fig. S3‒S4). Leave-one-out plots assessed the impact of individual IVs on overall MR estimates (Fig. S5‒S6).

**Table 2:** Heterogeneity and horizontal pleiotropy in disease-hearing loss associations.

Exposure	Outcome	Heterogeneity		Pleiotropy	
		*Q* statistic (IVW)	p-value	MR-Egger Intercept	p-value
Multiple sclerosis	Mixed conductive and sensorineural hearing loss	38.24976	0.012047	−0.00053	0.969099
Multiple sclerosis	Sensorineural hearing loss	57.89601	2.64E-05	−0.00323	0.591836
Ankylosing spondylitis	Sensorineural hearing loss	38.37683	0.031711	−0.00839	0.006593
Type 1 diabetes	Sensorineural hearing loss	58.12776	0.008334	−0.00016	0.963591
Sjogren's syndrome	Conductive hearing loss (unspecified)	16.01283	0.024999	−0.08927	0.129355
Rheumatoid arthritis	Mixed conductive and sensorineural hearing loss	36.85992	0.024507	0.015211	0.207839
Rheumatoid arthritis	Sensorineural hearing loss	69.08753	9.20E-07	0.002989	0.618215
Systemic lupus erythematosus	Mixed conductive and sensorineural hearing loss	41.75057	0.000135	−0.03432	0.290603
Systemic lupus erythematosus	Sensorineural hearing loss	161.3599	3.78E-27	−0.0171	0.458032
Crohn's disease	Mixed conductive and sensorineural hearing loss	66.50807	0.048573	0.00364	0.739962

MR-PRESSO identified outliers in ten associations, including MS-MHL, MS-SNHL, AS-SNHL, T1D-SNHL, SS-CHL, RA-MHL, RA-SHL, SLE-MHL, SLE-SNHL, and UC-SNHL associations (Table 3). The complete results of the MR-PRESSO analysis analysis are shown in Table S7. Among these, only AS-SNHL exhibited a significant association in the initial IVW analysis. After removing outliers (except for T1D-SNHL and SS-CHL with unavailable outlier numbers), IVW results remained significant for AS-SNHL (OR = 1.1722, 95% CI 1.0941–1.2558, p < 0.00001), and no additional significant associations were found (Table 4). The MR analysis results after outlier removal are shown in Table S8.

**Table 3:** Outlier detection and horizontal pleiotropy assessment using MR-PRESSO.

Exposure	Outcome	Raw		Outlier corrected		Global p	Number of outliers	Distortion p
		OR (CI%)	p	OR (95% CI)	p			
Multiple sclerosis	Mixed conductive and sensorineural hearing loss	1.0031 (0.9541–1.0546)	0.904912	0.9914 (0.9477–1.0371)	0.711825	0.0232	1	0.1745
Multiple sclerosis	Sensorineural hearing loss	1.017 (0.9949–1.0395)	0.147015	1.0158 (0.997–1.0349)	0.115667	2.00E-04	2	0.8785
Ankylosing spondylitis	Sensorineural hearing loss	1.1903 (1.1104–1.276)	5.20E-05	NA (NA‒NA)	NA	0.047	NA	NA
Type 1 diabetes	Sensorineural hearing loss	1.0035 (0.9855–1.0218)	0.710001	NA (NA‒NA)	NA	0.0112	NA	NA
Sjogren's syndrome	Conductive hearing loss(unspecified)	1.0098 (0.9344–1.0914)	0.811777	NA (NA‒NA)	NA	0.0411	NA	NA
Rheumatoid arthritis	Mixed conductive and sensorineural hearing loss	0.9824 (0.9098–1.0609)	0.655393	1.0573 (0.9552–1.1702)	0.293919	0.0387	1	0.3157
Rheumatoid arthritis	Sensorineural hearing loss	0.975 (0.9387–1.0127)	0.203298	0.9775 (0.9462–1.0099)	0.185432	2.00E-04	2	0.6675
Systemic lupus erythematosus	Mixed conductive and sensorineural hearing loss	1.0469 (0.9806–1.1178)	0.191315	0.9879 (0.9574–1.0194)	0.459748	<5e-04	1	0.002
Systemic lupus erythematosus	Sensorineural hearing loss	1.0336 (0.9871–1.0823)	0.181131	0.9953 (0.9827–1.0081)	0.486091	<5e-04	2	<5e-04
Ulcerative colitis	Sensorineural hearing loss	1.0171 (0.9814–1.0542)	0.363372	1.0171 (0.9901–1.0448)	0.232842	0.0228	2	0.9979

**Table 4:** MR analysis of disease-hearing loss associations post-outlier removal.

Exposure	Outcome	N.SNPs	Methods	OR (95% CI)	p
Multiple sclerosis	Mixed conductive and sensorineural hearing loss	21	Inverse variance weighted	0.9914 (0.9477–1.0371)	0.7079
Multiple sclerosis	Sensorineural hearing loss	20	Inverse variance weighted	1.0158 (0.997–1.0349)	0.0992
Ankylosing spondylitis	Sensorineural hearing loss	22	Inverse variance weighted	1.1722 (1.0941–1.2558)	<0.00001
Rheumatoid arthritis	Mixed conductive and sensorineural hearing loss	22	Inverse variance weighted	1.0463 (0.9422–1.1619)	0.3977
Rheumatoid arthritis	Sensorineural hearing loss	21	Inverse variance weighted	0.9682 (0.9367–1.0008)	0.0555
Systemic lupus erythematosus	Mixed conductive and sensorineural hearing loss	14	Inverse variance weighted	0.9879 (0.9467–1.0308)	0.5745
Systemic lupus erythematosus	Sensorineural hearing loss	13	Inverse variance weighted	0.9953 (0.9795–1.0114)	0.5673
Ulcerative colitis	Sensorineural hearing loss	17	Inverse variance weighted	1.0197 (0.9909–1.0493)	0.1826

After outlier removal, significant heterogeneity persisted for MS-SNHL (*Q* = 35.36663, p = 0.012612), while both heterogeneity and horizontal pleiotropy were resolved for AS-SNHL (Table S9). In the MR-PRESSO analysis, the global p-value for MS-SNHL was less than 0.05, indicating potential pleiotropy. However, the number of outliers was unavailable, and the association was not influenced by horizontal pleiotropy. For all other analyses, neither horizontal pleiotropy nor significant heterogeneity was detected (Table S10).

## Discussion

This study provides evidence of causal relationships between ADs and HLs using MR analysis. Notable associations included AS-SNHL, AS-MHL, AS-CHL, AS-SIHL, SLE-CHL, UC-MHL, MS-SIHL, and CD-CHL and CD-SIHL. These results highlight the role of immune dysregulation in the pathogenesis of HL.

The association between AS and multiple HL subtypes highlights its potential role in auditory dysfunction. AS is a chronic inflammatory disease primarily affecting the axial skeleton, characterized by excessive immune activation, pro-inflammatory cytokine release, and systemic inflammation, which can contribute to vascular damage and multisystem involvement, including the auditory system.^28^ A systematic review and meta-analysis reported a 42.4% prevalence of HL in patients with AS, with significantly higher odds of HL and impaired hearing thresholds compared to individuals without AS.^29^ HL occurs when damage to the auditory system disrupts the function of inner ear cells and associated neural structures due to inflammation, oxidative stress, or vascular impairment.^30^ Pro-inflammatory cytokines elevated in AS, such as TNF-α and IL-6,^31^ disrupt cochlear microcirculation, leading to ischemic damage and impaired auditory function.^32^ Chronic inflammation in AS increases oxidative stress,^33^ which damages inner ear cells and contributes to HL. Vascular abnormalities frequently associated with AS, such as vasculitis and endothelial dysfunction,^34^ compromise blood flow to the cochlea, exacerbating auditory damage.^35^ These mechanisms provide a plausible explanation for the broad AS-HL associations observed in our study. Clinically, the robust AS-HL association warrants attention to auditory function in AS patients, particularly those with severe systemic inflammation or prolonged disease duration. Routine auditory monitoring may help identify early signs of HL and mitigate its impact on quality of life.

Following AS, Inflammatory Bowel Diseases (IBD), including CD and UC, were the second most frequently associated ADs with HL, demonstrating significant relationships with MHL, CHL, and SIHL. HL associated with CD may result from neuropathy of the cochlear nerve due to vitamin B1 deficiency, a common complication in CD caused by malnutrition, vomiting, or chronic inflammation.^36^ Additionally, SNHL in UC may be driven by autoimmune inner ear disease, immune-mediated damage to the cochlea, systemic inflammation, or ototoxic effects of medications commonly used in IBD treatment.^37^ Furthermore, mutations in the STXBP3 gene, implicated in very early onset IBD, disrupt epithelial cell polarization and immune regulation, which may contribute to bilateral SNHL in IBD, highlighting a genetic component to this association.^38^ Subclinical cochlear involvement, often beginning at high frequencies, has been documented in pediatric IBD patients and is proposed to result from vasculitis and immune complex deposition in cochlear vessels as additional mechanisms for HL.^39^ These findings underscore the importance of implementing early auditory screening and monitoring strategies in IBD patients with pediatric onset or severe systemic inflammation to prevent or mitigate HL progression effectively.

Significant associations were also found for SLE-CHL and MS-SIHL. SLE causes HL predominantly through immune complex deposition in the stria vascularis and endolymphatic sacs, leading to ischemia and cytotoxic damage to cochlear hair cells, with secondary SS further exacerbating the risk.^40^ Similarly, MS-SIHL may result from neuroinflammatory and neurodegenerative processes characteristic of MS, including oxidative stress, mitochondrial dysfunction, and demyelination within auditory pathways. The accumulation of toxic tau proteins in MS further disrupts neuronal function, potentially impairing auditory signal transmission and contributing to SNHL.^41^ Both SLE and MS involve inflammatory mechanisms that impair cochlear function, highlighting the need for targeted approaches to manage HL in these conditions.

This study provides novel evidence of robust AD-HL associations using MR methods. However, the relatively smaller sample sizes for certain conditions, such as SLE and less common HL subtypes may reduce statistical power. Additionally, the possibility of residual pleiotropy and confounding remains despite sensitivity analyses. The lack of longitudinal data further limits the ability to evaluate the temporal dynamics and potential reversibility of HL. Addressing these issues in future studies through larger datasets, refined statistical approaches, and longitudinal designs will enhance the robustness and clinical applicability of the findings.

## Conclusion

This study identified significant causal associations between ADs and specific subtypes of HL. These findings underscore the role of immune dysregulation in the pathogenesis of auditory dysfunction and highlight the need for early audiological assessment and targeted therapeutic strategies in AD management to mitigate HL and improve patient outcomes.

## ORCID ID

Yutong Huang: 0000-0002-0906-687X

Shengli Gao: 0000-0002-3523-2333

Renzhong Luo: 0000-0003-1521-4602

## Authors’ contributions

Yutong Huang carried out the studies, participated in collecting data, performed the statistical analysis and drafted the manuscript. Renzhong Luo and Shengli Gao participated in its design. All authors read and approved the final manuscript.

## Funding

This study was supported by supported by scientific research foundation for Ph.D of Guangzhou Women and Children's Medical Center, 10.13039/100009659Guangzhou Medical University (2021BS027, Applications of Electrically Evoked Cortical Auditory Evoked Potentials in Cochlear Implant), basci and applied basic research program of Guangzhou Municipal Science and Technology Bureau (2023A04J1240, Applications of Electrically Evoked Cortical Auditory Evoked Potentials in Postoperative Evaluation for Pediatric Cochlear), and Science and Technology Project of Guangzhou (2023A03J0909, etiology and genetic analysis of severe sensorineural hearing loss in children in the Pearl River Delta).

## Conflicts of interest

The authors declare that the research was conducted in the absence of any commercial or financial relationships that could be construed as a potential conflict of interest.

## Data availability statement

All data generated or analysed during this study are included in this published article and its Supplementary Information File.
