# Supplementary material for: Exploring causal associations between autoimmune diseases and hearing loss: a mendelian randomization study
Source: Braz J Otorhinolaryngol. 2026 May 28;92(5):101837. doi: 10.1016/j.bjorl.2026.101837 (PMC13240669; doi:10.1016/j.bjorl.2026.101837)

BJORL-D-25-00025_Supplementary Material

**Table S1** GWAS data sources.

| **Trait** | **GWAS ID** | **Sample size (Case/Control)** | **SNP counts** |
| --- | --- | --- | --- |
| **Exposure** |  |  |  |
| Ankylosing spondylitis | ebi-a-GCST005529 | 9069/226473 | 99,962 |
| Rheumatoid arthritis | ebi-a-GCST90018910 | 8255/409,001 | 24,175,266 |
| Sjogren's syndrome | ebi-a-GCST90013879 | 407,746 | 11,039,117 |
| Systemic lupus erythematosus | ebi-a-GCST90018917 | 647/482,264 | 24,198,877 |
| multiple sclerosis | ebi-a-GCST003566 | 4888/10395 | 7,910,365 |
| Type 1 diabetes | ebi-a-GCST005536 | 6683/12173 | 101,101 |
| Ulcerative colitis | ebi-a-GCST90018933 | 5371/432561 | 24,187,301 |
| Crohn's disease | ieu-a-30 | 5956/14927 | 12,276,506 |
| **Outcome** |  |  |  |
| Conductive hearing loss, unspecified | H8_HL_CON_NAS | 2918/397865 | NA |
| Sudden idiopathic hearing loss | H8_HL_IDIOP | 3487/397865 | NA |
| Mixed conductive and sensorineural hearing loss | H8_HL_MIX_NAS | 4292/397865 | NA |
| Sensorineural hearing loss | H8_HL_SEN_NAS | 39620/397865 | NA |

**Table S2** IV characteristics.

| **Exposure** | **Outcome** | **Number of IVs** | **F-statistic (mean)** | **F-statistic (min)** | **F-statistic (max)** | **Unmatched SNPs** |
| --- | --- | --- | --- | --- | --- | --- |
| Ulcerative colitis | Sudden idiopathic hearing loss | 21 | 49.81962697 | 30.38415 | 152.4894 | rs139523312 |
| Ulcerative colitis | Sensorineural hearing loss | 21 | 49.81962697 | 30.38415 | 152.4894 | rs139523312 |
| Ulcerative colitis | Mixed conductive and sensorineural hearing loss | 21 | 49.81962697 | 30.38415 | 152.4894 | rs139523312 |
| Ulcerative colitis | Conductive hearing loss(unspecified) | 21 | 49.81962697 | 30.38415 | 152.4894 | rs139523312 |
| Type 1 diabetes | Sudden idiopathic hearing loss | 36 | 95.33210544 | 29.82542 | 892.0933 | rs2045258 |
| Type 1 diabetes | Sensorineural hearing loss | 36 | 95.33210544 | 29.82542 | 892.0933 | rs2045258 |
| Type 1 diabetes | Mixed conductive and sensorineural hearing loss | 36 | 95.33210544 | 29.82542 | 892.0933 | rs2045258 |
| Type 1 diabetes | Conductive hearing loss(unspecified) | 36 | 95.33210544 | 29.82542 | 892.0933 | rs2045258 |
| Systemic lupus erythematosus | Sudden idiopathic hearing loss | 15 | 30.49109698 | 20.85171 | 74.80785 | rs1131114 |
| Systemic lupus erythematosus | Sensorineural hearing loss | 15 | 30.49109698 | 20.85171 | 74.80785 | rs1131114 |
| Systemic lupus erythematosus | Mixed conductive and sensorineural hearing loss | 15 | 30.49109698 | 20.85171 | 74.80785 | rs1131114 |
| Systemic lupus erythematosus | Conductive hearing loss(unspecified) | 15 | 30.49109698 | 20.85171 | 74.80785 | rs1131114 |
| Sjogren's syndrome | Sudden idiopathic hearing loss | 10 | 32.47957211 | 18.92259 | 70.00565 | rs200801362, rs28631719 |
| Sjogren's syndrome | Sensorineural hearing loss | 10 | 32.47957211 | 18.92259 | 70.00565 | rs200801362, rs28631719 |
| Sjogren's syndrome | Mixed conductive and sensorineural hearing loss | 10 | 32.47957211 | 18.92259 | 70.00565 | rs200801362, rs28631719 |
| Sjogren's syndrome | Conductive hearing loss (unspecified) | 10 | 32.47957211 | 18.92259 | 70.00565 | rs200801362, rs28631719 |
| Rheumatoid arthritis | Sudden idiopathic hearing loss | 25 | 134.7687671 | 30.72831 | 1307.132 | rs56376587, rs1611318 |
| Rheumatoid arthritis | Sensorineural hearing loss | 25 | 134.7687671 | 30.72831 | 1307.132 | rs56376587, rs1611318 |
| Rheumatoid arthritis | Mixed conductive and sensorineural hearing loss | 25 | 134.7687671 | 30.72831 | 1307.132 | rs56376587, rs1611318 |
| Rheumatoid arthritis | Conductive hearing loss (unspecified) | 25 | 134.7687671 | 30.72831 | 1307.132 | rs56376587, rs1611318 |
| Multiple sclerosis | Sudden idiopathic hearing loss | 22 | 100.0883704 | 29.88154 | 1069.45 | rs34768512 |
| Multiple sclerosis | Sensorineural hearing loss | 22 | 100.0883704 | 29.88154 | 1069.45 | rs34768512 |
| Multiple sclerosis | Mixed conductive and sensorineural hearing loss | 22 | 100.0883704 | 29.88154 | 1069.45 | rs34768512 |
| Multiple sclerosis | Conductive hearing loss (unspecified) | 22 | 100.0883704 | 29.88154 | 1069.45 | rs34768512 |
| Crohn's disease | Sudden idiopathic hearing loss | 52 | 61.1271167 | 29.96719 | 272.9143 | rs7276302 |
| Crohn's disease | Sensorineural hearing loss | 52 | 61.1271167 | 29.96719 | 272.9143 | rs7276302 |
| Crohn's disease | Mixed conductive and sensorineural hearing loss | 52 | 61.1271167 | 29.96719 | 272.9143 | rs7276302 |
| Crohn's disease | Conductive hearing loss (unspecified) | 52 | 61.1271167 | 29.96719 | 272.9143 | rs7276302 |
| Ankylosing spondylitis | Sudden idiopathic hearing loss | 26 | 153.7186544 | 29.82734 | 1319.059 | rs743479 |
| Ankylosing spondylitis | Sensorineural hearing loss | 26 | 153.7186544 | 29.82734 | 1319.059 | rs743479 |
| Ankylosing spondylitis | Mixed conductive and sensorineural hearing loss | 26 | 153.7186544 | 29.82734 | 1319.059 | rs743479 |
| Ankylosing spondylitis | Conductive hearing loss (unspecified) | 26 | 153.7186544 | 29.82734 | 1319.059 | rs743479 |

**Table S3** List of proxy SNPs used in the study.

| **Original SNP** | **Proxy SNP** | **Exposure** | **Outcome** |
| --- | --- | --- | --- |
| rs1131114 | rs2853953 | Systemic lupus erythematosus | Sudden idiopathic hearing loss |
| rs1131114 | rs2853953 |  | Sensorineural hearing loss |
| rs1131114 | rs2853953 |  | Mixed conductive and sensorineural hearing loss |
| rs1131114 | rs2853953 |  | Conductive hearing loss(unspecified) |
| rs56376587 | rs8096658 | Rheumatoid arthritis | Sudden idiopathic hearing loss |
| rs56376587 | rs8096658 |  | Sensorineural hearing loss |
| rs56376587 | rs8096658 |  | Mixed conductive and sensorineural hearing loss |
| rs56376587 | rs8096658 |  | Conductive hearing loss(unspecified) |
| rs2045258 | rs9491624 | Type 1 diabetes | Sudden idiopathic hearing loss |
| rs2045258 | rs9491624 |  | Sensorineural hearing loss |
| rs2045258 | rs9491624 |  | Mixed conductive and sensorineural hearing loss |
| rs2045258 | rs9491624 |  | Conductive hearing loss(unspecified) |
| rs34768512 | rs34196875 | Multiple sclerosis | Sudden idiopathic hearing loss |
| rs34768512 | rs34196875 |  | Sensorineural hearing loss |
| rs34768512 | rs34196875 |  | Mixed conductive and sensorineural hearing loss |
| rs34768512 | rs34196875 |  | Conductive hearing loss(unspecified) |

**Table S4** List of palindromic SNPs.

| **Exposure** | **Outcome** | **Palindromic SNPs** |
| --- | --- | --- |
| Crohn's disease | Conductive hearing loss (unspecified) | rs12692254 |
| Crohn's disease | Mixed conductive and sensorineural hearing loss | rs12692254 |
| Crohn's disease | Sensorineural hearing loss | rs12692254 |
| Crohn's disease | Sudden idiopathic hearing loss | rs12692254 |
| Rheumatoid arthritis | Conductive hearing loss (unspecified) | rs3093017 |
| Rheumatoid arthritis | Mixed conductive and sensorineural hearing loss | rs3093017 |
| Rheumatoid arthritis | Sensorineural hearing loss | rs3093017 |
| Rheumatoid arthritis | Sudden idiopathic hearing loss | rs3093017 |
| Ulcerative colitis | Conductive hearing loss (unspecified) | rs4065985, rs7936434 |
| Ulcerative colitis | Mixed conductive and sensorineural hearing loss | rs4065985, rs7936434 |
| Ulcerative colitis | Sensorineural hearing loss | rs4065985, rs7936434 |
| Ulcerative colitis | Sudden idiopathic hearing loss | rs4065985, rs7936434 |

**Table S5** The complete results of the MR analysis.

| **Exposure** | **Outcome** | **N.SNPs** | **Methods** | **OR (95% CI)** | **p** |
| --- | --- | --- | --- | --- | --- |
| Multiple sclerosis | Conductive hearing loss (unspecified) | 22 | Inverse variance weighted | 1.0198 (0.9692‒1.0731) | 0.4495 |
| Multiple sclerosis | Conductive hearing loss (unspecified) | 22 | MR Egger | 1.026 (0.9349‒1.1259) | 0.5949 |
| Multiple sclerosis | Conductive hearing loss (unspecified) | 22 | Weighted median | 1.0024 (0.9406‒1.0682) | 0.9417 |
| Multiple sclerosis | Conductive hearing loss (unspecified) | 22 | Weighted mode | 1.0153 (0.9574‒1.0768) | 0.6171 |
| Multiple sclerosis | Mixed conductive and sensorineural hearing loss | 22 | Inverse variance weighted | 1.0031 (0.9541‒1.0546) | 0.9038 |
| Multiple sclerosis | Mixed conductive and sensorineural hearing loss | 22 | MR Egger | 1.0046 (0.9169‒1.1006) | 0.9223 |
| Multiple sclerosis | Mixed conductive and sensorineural hearing loss | 22 | Weighted median | 0.9797 (0.9289‒1.0332) | 0.4487 |
| Multiple sclerosis | Mixed conductive and sensorineural hearing loss | 22 | Weighted mode | 0.9951 (0.9381‒1.0556) | 0.8725 |
| Multiple sclerosis | Sensorineural hearing loss | 22 | Inverse variance weighted | 1.017 (0.9949‒1.0395) | 0.1321 |
| Multiple sclerosis | Sensorineural hearing loss | 22 | MR Egger | 1.0263 (0.9864‒1.0679) | 0.2144 |
| Multiple sclerosis | Sensorineural hearing loss | 22 | Weighted median | 1.021 (1.0014‒1.0409) | 0.0351 |
| Multiple sclerosis | Sensorineural hearing loss | 22 | Weighted mode | 1.0217 (1.0015‒1.0424) | 0.0474 |
| Multiple sclerosis | Sudden idiopathic hearing loss | 22 | Inverse variance weighted | 1.0494 (1.0072‒1.0934) | 0.0213 |
| Multiple sclerosis | Sudden idiopathic hearing loss | 22 | MR Egger | 0.9842 (0.9147‒1.059) | 0.6747 |
| Multiple sclerosis | Sudden idiopathic hearing loss | 22 | Weighted median | 1.0027 (0.9462‒1.0625) | 0.928 |
| Multiple sclerosis | Sudden idiopathic hearing loss | 22 | Weighted mode | 0.9937 (0.9327‒1.0586) | 0.8461 |
| Ankylosing spondylitis | Conductive hearing loss (unspecified) | 25 | Inverse variance weighted | 1.2832 (1.0643‒1.5472) | 0.009 |
| Ankylosing spondylitis | Conductive hearing loss (unspecified) | 25 | MR Egger | 1.161 ( 0.8858‒1.5217 ) | 0.2908 |
| Ankylosing spondylitis | Conductive hearing loss (unspecified) | 25 | Weighted median | 1.1407 (0.8801‒1.4784) | 0.3198 |
| Ankylosing spondylitis | Conductive hearing loss (unspecified) | 25 | Weighted mode | 1.1588 (0.9328‒1.4394) | 0.1954 |
| Ankylosing spondylitis | Mixed conductive and sensorineural hearing loss | 25 | Inverse variance weighted | 1.5994 (1.3696‒1.8678) | 0 |
| Ankylosing spondylitis | Mixed conductive and sensorineural hearing loss | 25 | MR Egger | 1.8096 (1.4558‒2.2494) | 0 |
| Ankylosing spondylitis | Mixed conductive and sensorineural hearing loss | 25 | Weighted median | 1.7327 (1.4301‒2.0993) | 0 |
| Ankylosing spondylitis | Mixed conductive and sensorineural hearing loss | 25 | Weighted mode | 1.6615 (1.382‒1.9974) | 0 |
| Ankylosing spondylitis | Sensorineural hearing loss | 25 | Inverse variance weighted | 1.1903 (1.1104‒1.276) | 0 |
| Ankylosing spondylitis | Sensorineural hearing loss | 25 | MR Egger | 1.3093 (1.2004‒1.4282) | 0 |
| Ankylosing spondylitis | Sensorineural hearing loss | 25 | Weighted median | 1.238 (1.1507‒1.3319) | 0 |
| Ankylosing spondylitis | Sensorineural hearing loss | 25 | Weighted mode | 1.2399 (1.1648‒1.3198) | 0 |
| Ankylosing spondylitis | Sudden idiopathic hearing loss | 25 | Inverse variance weighted | 1.481 (1.22‒1.798) | 1.00E-04 |
| Ankylosing spondylitis | Sudden idiopathic hearing loss | 25 | MR Egger | 1.3855 (1.0454‒1.8363) | 0.0329 |
| Ankylosing spondylitis | Sudden idiopathic hearing loss | 25 | Weighted median | 1.3923 (1.1021‒1.7589) | 0.0055 |
| Ankylosing spondylitis | Sudden idiopathic hearing loss | 25 | Weighted mode | 1.4048 (1.1249‒1.7543) | 0.0062 |
| Type 1 diabetes | Conductive hearing loss (unspecified) | 36 | Inverse variance weighted | 1.0128 (0.9576‒1.0711) | 0.6569 |
| Type 1 diabetes | Conductive hearing loss (unspecified) | 36 | MR Egger | 0.9952 (0.8995‒1.1011) | 0.9265 |
| Type 1 diabetes | Conductive hearing loss (unspecified) | 36 | Weighted median | 0.9845 (0.9185‒1.0553) | 0.66 |
| Type 1 diabetes | Conductive hearing loss (unspecified) | 36 | Weighted mode | 0.9875 (0.9228‒1.0567) | 0.718 |
| Type 1 diabetes | Mixed conductive and sensorineural hearing loss | 36 | Inverse variance weighted | 0.9967 (0.9535‒1.0419) | 0.8846 |
| Type 1 diabetes | Mixed conductive and sensorineural hearing loss | 36 | MR Egger | 0.9733 (0.8987‒1.0541) | 0.5104 |
| Type 1 diabetes | Mixed conductive and sensorineural hearing loss | 36 | Weighted median | 0.9783 (0.9241‒1.0356) | 0.4491 |
| Type 1 diabetes | Mixed conductive and sensorineural hearing loss | 36 | Weighted mode | 0.9789 (0.9238‒1.0372) | 0.4749 |
| Type 1 diabetes | Sensorineural hearing loss | 36 | Inverse variance weighted | 1.0035 (0.9855‒1.0218) | 0.7077 |
| Type 1 diabetes | Sensorineural hearing loss | 36 | MR Egger | 1.0041 (0.9718‒1.0375) | 0.8079 |
| Type 1 diabetes | Sensorineural hearing loss | 36 | Weighted median | 1.0013 (0.9787‒1.0244) | 0.9112 |
| Type 1 diabetes | Sensorineural hearing loss | 36 | Weighted mode | 1.0002 (0.9791‒1.0217) | 0.9881 |
| Type 1 diabetes | Sudden idiopathic hearing loss | 36 | Inverse variance weighted | 0.9945 (0.9514‒1.0396) | 0.8081 |
| Type 1 diabetes | Sudden idiopathic hearing loss | 36 | MR Egger | 1.0125 (0.9347‒1.0968) | 0.7626 |
| Type 1 diabetes | Sudden idiopathic hearing loss | 36 | Weighted median | 0.9931 (0.9294‒1.0611) | 0.8367 |
| Type 1 diabetes | Sudden idiopathic hearing loss | 36 | Weighted mode | 1.0014 (0.9455‒1.0607) | 0.9611 |
| Sjogren's syndrome | Conductive hearing loss(unspecified) | 8 | Inverse variance weighted | 1.0098 (0.9344‒1.0914) | 0.8047 |
| Sjogren's syndrome | Conductive hearing loss (unspecified) | 8 | MR Egger | 1.191 (0.9788‒1.4491) | 0.1314 |
| Sjogren's syndrome | Conductive hearing loss (unspecified) | 8 | Weighted median | 1.0477 (0.9745‒1.1263) | 0.2071 |
| Sjogren's syndrome | Conductive hearing loss (unspecified) | 8 | Weighted mode | 1.0564 (0.9763‒1.1431) | 0.215 |
| Sjogren's syndrome | Mixed conductive and sensorineural hearing loss | 8 | Inverse variance weighted | 1.0124 (0.9679‒1.0589) | 0.5912 |
| Sjogren's syndrome | Mixed conductive and sensorineural hearing loss | 8 | MR Egger | 1.1111 (0.9827‒1.2563) | 0.1438 |
| Sjogren's syndrome | Mixed conductive and sensorineural hearing loss | 8 | Weighted median | 0.9992 (0.9419‒1.06) | 0.979 |
| Sjogren's syndrome | Mixed conductive and sensorineural hearing loss | 8 | Weighted mode | 0.9967 (0.9177‒1.0825) | 0.9393 |
| Sjogren's syndrome | Sensorineural hearing loss | 8 | Inverse variance weighted | 1.01 (0.9932‒1.0271) | 0.2463 |
| Sjogren's syndrome | Sensorineural hearing loss | 8 | MR Egger | 1.035 (0.9867‒1.0857) | 0.2079 |
| Sjogren's syndrome | Sensorineural hearing loss | 8 | Weighted median | 1.0182 (0.9988‒1.0379) | 0.0662 |
| Sjogren's syndrome | Sensorineural hearing loss | 8 | Weighted mode | 1.0192 (0.9949‒1.0442) | 0.1656 |
| Sjogren's syndrome | Sudden idiopathic hearing loss | 8 | Inverse variance weighted | 0.9677 (0.923‒1.0145) | 0.173 |
| Sjogren's syndrome | Sudden idiopathic hearing loss | 8 | MR Egger | 1.0241 (0.8939‒1.1733) | 0.7431 |
| Sjogren's syndrome | Sudden idiopathic hearing loss | 8 | Weighted median | 0.9633 (0.9061‒1.0242) | 0.2319 |
| Sjogren's syndrome | Sudden idiopathic hearing loss | 8 | Weighted mode | 0.9576 (0.8703‒1.0536) | 0.4037 |
| Rheumatoid arthritis | Conductive hearing loss (unspecified) | 23 | Inverse variance weighted | 0.9869 (0.9137‒1.0659) | 0.7374 |
| Rheumatoid arthritis | Conductive hearing loss (unspecified) | 23 | MR Egger | 0.9391 (0.8343‒1.0571) | 0.3102 |
| Rheumatoid arthritis | Conductive hearing loss (unspecified) | 23 | Weighted median | 0.9329 (0.8443‒1.0308) | 0.1723 |
| Rheumatoid arthritis | Conductive hearing loss (unspecified) | 23 | Weighted mode | 0.9403 (0.8546‒1.0347) | 0.2203 |
| Rheumatoid arthritis | Mixed conductive and sensorineural hearing loss | 23 | Inverse variance weighted | 0.9755 (0.9028‒1.054) | 0.5297 |
| Rheumatoid arthritis | Mixed conductive and sensorineural hearing loss | 23 | MR Egger | 0.9193 (0.8173‒1.034) | 0.1752 |
| Rheumatoid arthritis | Mixed conductive and sensorineural hearing loss | 23 | Weighted median | 0.9233 (0.8549‒0.9971) | 0.042 |
| Rheumatoid arthritis | Mixed conductive and sensorineural hearing loss | 23 | Weighted mode | 0.9168 (0.8465‒0.9929) | 0.0441 |
| Rheumatoid arthritis | Sensorineural hearing loss | 23 | Inverse variance weighted | 0.971 (0.935‒1.0085) | 0.1277 |
| Rheumatoid arthritis | Sensorineural hearing loss | 23 | MR Egger | 0.9597 (0.9044‒1.0185) | 0.1898 |
| Rheumatoid arthritis | Sensorineural hearing loss | 23 | Weighted median | 0.945 (0.9176‒0.9733) | 2.00E-04 |
| Rheumatoid arthritis | Sensorineural hearing loss | 23 | Weighted mode | 0.9425 (0.9177‒0.968) | 3.00E-04 |
| Rheumatoid arthritis | Sudden idiopathic hearing loss | 23 | Inverse variance weighted | 1.0003 (0.9269‒1.0795) | 0.9943 |
| Rheumatoid arthritis | Sudden idiopathic hearing loss | 23 | MR Egger | 1.0502 (0.934‒1.1808) | 0.4225 |
| Rheumatoid arthritis | Sudden idiopathic hearing loss | 23 | Weighted median | 0.9697 (0.8901‒1.0564) | 0.4812 |
| Rheumatoid arthritis | Sudden idiopathic hearing loss | 23 | Weighted mode | 0.9957 (0.9139‒1.0848) | 0.9228 |
| Systemic lupus erythematosus | Conductive hearing loss (unspecified) | 15 | Inverse variance weighted | 1.0593 (1.0116‒1.1092) | 0.0142 |
| Systemic lupus erythematosus | Conductive hearing loss (unspecified) | 15 | MR Egger | 1.0579 (0.9542‒1.1728) | 0.3042 |
| Systemic lupus erythematosus | Conductive hearing loss (unspecified) | 15 | Weighted median | 1.0669 (1.0026‒1.1352) | 0.0412 |
| Systemic lupus erythematosus | Conductive hearing loss (unspecified) | 15 | Weighted mode | 1.0816 (0.9797‒1.194) | 0.1427 |
| Systemic lupus erythematosus | Mixed conductive and sensorineural hearing loss | 15 | Inverse variance weighted | 1.0469 (0.9806‒1.1178) | 0.1697 |
| Systemic lupus erythematosus | Mixed conductive and sensorineural hearing loss | 15 | MR Egger | 1.1265 (0.9738‒1.3031) | 0.1329 |
| Systemic lupus erythematosus | Mixed conductive and sensorineural hearing loss | 15 | Weighted median | 1.0044 (0.9483‒1.0639) | 0.881 |
| Systemic lupus erythematosus | Mixed conductive and sensorineural hearing loss | 15 | Weighted mode | 0.9828 (0.8947‒1.0796) | 0.7233 |
| Systemic lupus erythematosus | Sensorineural hearing loss | 15 | Inverse variance weighted | 1.0336 (0.9871‒1.0823) | 0.1593 |
| Systemic lupus erythematosus | Sensorineural hearing loss | 15 | MR Egger | 1.0721 (0.9655‒1.1905) | 0.2152 |
| Systemic lupus erythematosus | Sensorineural hearing loss | 15 | Weighted median | 0.9969 (0.9761‒1.0181) | 0.7724 |
| Systemic lupus erythematosus | Sensorineural hearing loss | 15 | Weighted mode | 0.9959 ( 0.9684‒1.0242) | 0.7795 |
| Systemic lupus erythematosus | Sudden idiopathic hearing loss | 15 | Inverse variance weighted | 1.0159 ( 0.9739‒1.0597 ) | 0.4639 |
| Systemic lupus erythematosus | Sudden idiopathic hearing loss | 15 | MR Egger | 1.1022 (1.0029‒1.2115) | 0.0645 |
| Systemic lupus erythematosus | Sudden idiopathic hearing loss | 15 | Weighted median | 1.0409 ( 0.9773‒1.1087 ) | 0.2124 |
| Systemic lupus erythematosus | Sudden idiopathic hearing loss | 15 | Weighted mode | 1.0605 (0.9724‒1.1567) | 0.2057 |
| Ulcerative colitis | Conductive hearing loss(unspecified) | 18 | Inverse variance weighted | 1.0877 (0.9763‒1.2119) | 0.1271 |
| Ulcerative colitis | Conductive hearing loss (unspecified) | 18 | MR Egger | 1.0361 ( 0.6984‒1.537) | 0.8625 |
| Ulcerative colitis | Conductive hearing loss (unspecified) | 18 | Weighted median | 1.0997 (0.9572‒1.2634) | 0.1797 |
| Ulcerative colitis | Conductive hearing loss (unspecified) | 18 | Weighted mode | 1.1833 (0.9456‒1.4808) | 0.1595 |
| Ulcerative colitis | Mixed conductive and sensorineural hearing loss | 18 | Inverse variance weighted | 1.0907 (1.0027‒1.1865) | 0.0431 |
| Ulcerative colitis | Mixed conductive and sensorineural hearing loss | 18 | MR Egger | 1.1379 (0.8389‒1.5436) | 0.4185 |
| Ulcerative colitis | Mixed conductive and sensorineural hearing loss | 18 | Weighted median | 1.0752 ( 0.9658‒1.1969 ) | 0.1854 |
| Ulcerative colitis | Mixed conductive and sensorineural hearing loss | 18 | Weighted mode | 1.0849 (0.9103‒1.293) | 0.3754 |
| Ulcerative colitis | Sensorineural hearing loss | 18 | Inverse variance weighted | 1.0283 (0.9951‒1.0627) | 0.0954 |
| Ulcerative colitis | Sensorineural hearing loss | 18 | MR Egger | 1.0699 (0.9513‒1.2033) | 0.2762 |
| Ulcerative colitis | Sensorineural hearing loss | 18 | Weighted median | 1.0293 (0.9889‒1.0714) | 0.1575 |
| Ulcerative colitis | Sensorineural hearing loss | 18 | Weighted mode | 1.0204 (0.9498‒1.0962) | 0.5885 |
| Ulcerative colitis | Sudden idiopathic hearing loss | 18 | Inverse variance weighted | 1.0112 (0.9156‒1.1167) | 0.8263 |
| Ulcerative colitis | Sudden idiopathic hearing loss | 18 | MR Egger | 0.8733 (0.6103‒1.2495) | 0.4692 |
| Ulcerative colitis | Sudden idiopathic hearing loss | 18 | Weighted median | 0.9501 (0.8446‒1.0687) | 0.3935 |
| Ulcerative colitis | Sudden idiopathic hearing loss | 18 | Weighted mode | 0.9109 (0.766‒1.0833) | 0.3063 |
| Crohn's disease | Conductive hearing loss (unspecified) | 50 | Inverse variance weighted | 1.0529 (1.0074‒1.1005) | 0.0222 |
| Crohn's disease | Conductive hearing loss (unspecified) | 50 | MR Egger | 1.1061 (0.9897‒1.2361) | 0.0817 |
| Crohn's disease | Conductive hearing loss (unspecified) | 50 | Weighted median | 1.0693 (1.0001‒1.1433) | 0.0497 |
| Crohn's disease | Conductive hearing loss (unspecified) | 50 | Weighted mode | 1.0781 (0.9839‒1.1814) | 0.1134 |
| Crohn's disease | Mixed conductive and sensorineural hearing loss | 50 | Inverse variance weighted | 1.0118 (0.9707‒1.0547) | 0.5787 |
| Crohn's disease | Mixed conductive and sensorineural hearing loss | 50 | MR Egger | 0.9953 (0.8959‒1.1058) | 0.9308 |
| Crohn's disease | Mixed conductive and sensorineural hearing loss | 50 | Weighted median | 1.0067 (0.949‒1.0679) | 0.8254 |
| Crohn's disease | Mixed conductive and sensorineural hearing loss | 50 | Weighted mode | 1.0248 (0.9438‒1.1128) | 0.5621 |
| Crohn's disease | Sensorineural hearing loss | 50 | Inverse variance weighted | 1.0091 (0.9951‒1.0233) | 0.2021 |
| Crohn's disease | Sensorineural hearing loss | 50 | MR Egger | 1.0157 (0.9804‒1.0523) | 0.3913 |
| Crohn's disease | Sensorineural hearing loss | 50 | Weighted median | 1.0074 (0.9875‒1.0277) | 0.4712 |
| Crohn's disease | Sensorineural hearing loss | 50 | Weighted mode | 1.0026 (0.9737‒1.0323) | 0.8635 |
| Crohn's disease | Sudden idiopathic hearing loss | 50 | Inverse variance weighted | 1.0597 (1.0177‒1.1034) | 0.005 |
| Crohn's disease | Sudden idiopathic hearing loss | 50 | MR Egger | 1.0365 (0.9356‒1.1483) | 0.4957 |
| Crohn's disease | Sudden idiopathic hearing loss | 50 | Weighted median | 1.0359 (0.977‒1.0983) | 0.2375 |
| Crohn's disease | Sudden idiopathic hearing loss | 50 | Weighted mode | 1.0302 (0.951‒1.1159) | 0.4694 |

**Table S6** The complete results of Heterogeneity and horizontal pleiotropy in disease-hearing loss associations.

| **Exposure** | **Outcome** | **Heterogeneity** | | **Pleiotropy** | |
| --- | --- | --- | --- | --- | --- |
|  |  | **Q statistic (IVW)** | **p-value** | **MR-Egger Intercept** | **p-value** |
| Multiple sclerosis | Conductive hearing loss (unspecified) | 27.19745 | 0.164413 | -0.00211 | 0.880478 |
| Multiple sclerosis | Mixed conductive and sensorineural hearing loss | 38.24976 | 0.012047 | -0.00053 | 0.969099 |
| Multiple sclerosis | Sensorineural hearing loss | 57.89601 | 2.64E-05 | -0.00323 | 0.591836 |
| Multiple sclerosis | Sudden idiopathic hearing loss | 15.76676 | 0.78261 | 0.022614 | 0.051061 |
| Ankylosing spondylitis | Conductive hearing loss (unspecified) | 22.031 | 0.577416 | 0.00873 | 0.325905 |
| Ankylosing spondylitis | Mixed conductive and sensorineural hearing loss | 24.95576 | 0.408168 | -0.01109 | 0.133537 |
| Ankylosing spondylitis | Sensorineural hearing loss | 38.37683 | 0.031711 | -0.00839 | 0.006593 |
| Ankylosing spondylitis | Sudden idiopathic hearing loss | 31.60903 | 0.136987 | 0.005945 | 0.524097 |
| Type 1 diabetes | Conductive hearing loss (unspecified) | 48.81965 | 0.060417 | 0.004498 | 0.684971 |
| Type 1 diabetes | Mixed conductive and sensorineural hearing loss | 44.66916 | 0.126771 | 0.006122 | 0.48525 |
| Type 1 diabetes | Sensorineural hearing loss | 58.12776 | 0.008334 | -0.00016 | 0.963591 |
| Type 1 diabetes | Sudden idiopathic hearing loss | 36.4151 | 0.402673 | -0.00461 | 0.599514 |
| Sjogren's syndrome | Conductive hearing loss (unspecified) | 16.01283 | 0.024999 | -0.08927 | 0.129355 |
| Sjogren's syndrome | Mixed conductive and sensorineural hearing loss | 7.751062 | 0.355056 | -0.05031 | 0.164498 |
| Sjogren's syndrome | Sensorineural hearing loss | 8.540017 | 0.287388 | -0.01325 | 0.325536 |
| Sjogren's syndrome | Sudden idiopathic hearing loss | 4.079662 | 0.770558 | -0.03064 | 0.417125 |
| Rheumatoid arthritis | Conductive hearing loss (unspecified) | 25.1536 | 0.289786 | 0.01273 | 0.292675 |
| Rheumatoid arthritis | Mixed conductive and sensorineural hearing loss | 36.85992 | 0.024507 | 0.015211 | 0.207839 |
| Rheumatoid arthritis | Sensorineural hearing loss | 69.08753 | 9.20E-07 | 0.002989 | 0.618215 |
| Rheumatoid arthritis | Sudden idiopathic hearing loss | 29.19952 | 0.139252 | -0.01247 | 0.297578 |
| Systemic lupus erythematosus | Conductive hearing loss (unspecified) | 10.17936 | 0.748956 | 0.000613 | 0.978148 |
| Systemic lupus erythematosus | Mixed conductive and sensorineural hearing loss | 41.75057 | 0.000135 | -0.03432 | 0.290603 |
| Systemic lupus erythematosus | Sensorineural hearing loss | 161.3599 | 3.78E-27 | -0.0171 | 0.458032 |
| Systemic lupus erythematosus | Sudden idiopathic hearing loss | 11.58321 | 0.639735 | -0.0381 | 0.080993 |
| Ulcerative colitis | Conductive hearing loss (unspecified) | 22.00844 | 0.184395 | 0.00761 | 0.80412 |
| Ulcerative colitis | Mixed conductive and sensorineural hearing loss | 19.43302 | 0.30425 | -0.00663 | 0.780108 |
| Ulcerative colitis | Sensorineural hearing loss | 23.23857 | 0.141644 | -0.0062 | 0.500302 |
| Ulcerative colitis | Sudden idiopathic hearing loss | 22.00125 | 0.184671 | 0.022877 | 0.415733 |
| Crohn's disease | Conductive hearing loss (unspecified) | 51.96386 | 0.359184 | -0.0109 | 0.348616 |
| Crohn's disease | Mixed conductive and sensorineural hearing loss | 66.50807 | 0.048573 | 0.00364 | 0.739962 |
| Crohn's disease | Sensorineural hearing loss | 58.84522 | 0.158385 | -0.00145 | 0.694419 |
| Crohn's disease | Sudden idiopathic hearing loss | 51.54197 | 0.37464 | 0.00489 | 0.64702 |

**Table S7** The complete results of MR-PRESSO analysis.

| **Exposure** | **Outcome** | **Raw** | | **Outlier corrected** | | **Global p** | **Number of outliers** | **Distortion p** |
| --- | --- | --- | --- | --- | --- | --- | --- | --- |
|  |  | **OR (95% CI)** | **p** | **OR (95% CI)** | **p** |  |  |  |
| Multiple sclerosis | Conductive hearing loss (unspecified) | 1.0198 (0.9692‒1.0731) | 0.457872 | NA (NA‒NA) | NA | 0.2009 | NA | NA |
| Multiple sclerosis | Mixed conductive and sensorineural hearing loss | 1.0031 (0.9541‒1.0546) | 0.904912 | 0.9914 (0.9477‒1.0371) | 0.711825 | 0.0232 | 1 | 0.1745 |
| Multiple sclerosis | Sensorineural hearing loss | 1.017 (0.9949‒1.0395) | 0.147015 | 1.0158 (0.997‒1.0349) | 0.115667 | 2.00E-04 | 2 | 0.8785 |
| Multiple sclerosis | Sudden idiopathic hearing loss | 1.0494 (1.0128‒1.0875) | 0.014721 | NA (NA‒NA) | NA | 0.5854 | NA | NA |
| Ankylosing spondylitis | Conductive hearing loss(unspecified) | 1.2832 (1.0727‒1.5351) | 0.011749 | NA (NA‒NA) | NA | 0.5435 | NA | NA |
| Ankylosing spondylitis | Mixed conductive and sensorineural hearing loss | 1.5994 (1.3696‒1.8678) | 4.00E-06 | NA (NA‒NA) | NA | 0.4496 | NA | NA |
| Ankylosing spondylitis | Sensorineural hearing loss | 1.1903 (1.1104‒1.276) | 5.20E-05 | NA (NA‒NA) | NA | 0.047 | NA | NA |
| Ankylosing spondylitis | Sudden idiopathic hearing loss | 1.481 (1.22‒1.798) | 0.000569 | NA (NA‒NA) | NA | 0.1518 | NA | NA |
| Type 1 diabetes | Conductive hearing loss (unspecified) | 1.0128 (0.9576‒1.0711) | 0.659605 | NA (NA‒NA) | NA | 0.0732 | NA | NA |
| Type 1 diabetes | Mixed conductive and sensorineural hearing loss | 0.9967 (0.9535‒1.0419) | 0.885439 | NA (NA‒NA) | NA | 0.148 | NA | NA |
| Type 1 diabetes | Sensorineural hearing loss | 1.0035 (0.9855‒1.0218) | 0.710001 | NA (NA‒NA) | NA | 0.0112 | NA | NA |
| Type 1 diabetes | Sudden idiopathic hearing loss | 0.9945 (0.9514‒1.0396) | 0.809525 | NA (NA‒NA) | NA | 0.4341 | NA | NA |
| Sjogren's syndrome | Conductive hearing loss (unspecified) | 1.0098 (0.9344‒1.0914) | 0.811777 | NA (NA‒NA) | NA | 0.0411 | NA | NA |
| Sjogren's syndrome | Mixed conductive and sensorineural hearing loss | 1.0124 (0.9679‒1.0589) | 0.607822 | NA (NA‒NA) | NA | 0.3616 | NA | NA |
| Sjogren's syndrome | Sensorineural hearing loss | 1.01 (0.9932‒1.0271) | 0.284338 | NA (NA‒NA) | NA | 0.3656 | NA | NA |
| Sjogren's syndrome | Sudden idiopathic hearing loss | 0.9677 (0.9334‒1.0032) | 0.117477 | NA (NA‒NA) | NA | 0.7748 | NA | NA |
| Rheumatoid arthritis | Conductive hearing loss (unspecified) | 0.9975 (0.9217‒1.0795) | 0.950993 | NA (NA‒NA) | NA | 0.1925 | NA | NA |
| Rheumatoid arthritis | Mixed conductive and sensorineural hearing loss | 0.9824 (0.9098‒1.0609) | 0.655393 | 1.0573 (0.9552‒1.1702) | 0.293919 | 0.0387 | 1 | 0.3157 |
| Rheumatoid arthritis | Sensorineural hearing loss | 0.975 (0.9387‒1.0127) | 0.203298 | 0.9775 (0.9462‒1.0099) | 0.185432 | 2.00E-04 | 2 | 0.6675 |
| Rheumatoid arthritis | Sudden idiopathic hearing loss | 1.0087 (0.9344‒1.0889) | 0.826462 | NA (NA‒NA) | NA | 0.1454 | NA | NA |
| Systemic lupus erythematosus | Conductive hearing loss (unspecified) | 1.0593 (1.0185‒1.1017) | 0.01223 | NA (NA‒NA) | NA | 0.761 | NA | NA |
| Systemic lupus erythematosus | Mixed conductive and sensorineural hearing loss | 1.0469 (0.9806‒1.1178) | 0.191315 | 0.9879 (0.9574‒1.0194) | 0.459748 | <5e-04 | 1 | 0.002 |
| Systemic lupus erythematosus | Sensorineural hearing loss | 1.0336 (0.9871‒1.0823) | 0.181131 | 0.9953 (0.9827‒1.0081) | 0.486091 | <5e-04 | 2 | <5e-04 |
| Systemic lupus erythematosus | Sudden idiopathic hearing loss | 1.0159 (0.9776‒1.0557) | 0.434161 | NA (NA‒NA) | NA | 0.626 | NA | NA |
| Ulcerative colitis | Conductive hearing loss (unspecified) | 1.0832 (0.9793‒1.1981) | 0.136979 | NA (NA‒NA) | NA | 0.24 | NA | NA |
| Ulcerative colitis | Mixed conductive and sensorineural hearing loss | 1.0891 (1.0039‒1.1816) | 0.054116 | NA (NA‒NA) | NA | 0.3066 | NA | NA |
| Ulcerative colitis | Sensorineural hearing loss | 1.0171 (0.9814‒1.0542) | 0.363372 | 1.0171 (0.9901‒1.0448) | 0.232842 | 0.0228 | 2 | 0.9979 |
| Ulcerative colitis | Sudden idiopathic hearing loss | 0.9944 (0.9023‒1.0958) | 0.910611 | NA (NA‒NA) | NA | 0.1537 | NA | NA |
| Crohn's disease | Conductive hearing loss (unspecified) | 1.0459 (1.0018‒1.092) | 0.046484 | NA (NA‒NA) | NA | 0.3487 | NA | NA |
| Crohn's disease | Mixed conductive and sensorineural hearing loss | 1.0135 (0.9738‒1.0548) | 0.513088 | NA (NA‒NA) | NA | 0.0615 | NA | NA |
| Crohn's disease | Sensorineural hearing loss | 1.0079 (0.9945‒1.0216) | 0.253818 | NA (NA‒NA) | NA | 0.1797 | NA | NA |
| Crohn's disease | Sudden idiopathic hearing loss | 1.0525 (1.0117‒1.095) | 0.014444 | NA (NA‒NA) | NA | 0.3533 | NA | NA |

**Table S8** MR analysis results after outlier removal.

| **Exposure** | **Outcome** | **N.SNPs** | **Methods** | **OR (95% CI)** | **p** |
| --- | --- | --- | --- | --- | --- |
| Multiple sclerosis | Mixed conductive and sensorineural hearing loss | 21 | Inverse variance weighted | 0.9914 (0.9477‒1.0371) | 0.7079 |
| Multiple sclerosis | Mixed conductive and sensorineural hearing loss | 21 | MR Egger | 1.0029 (0.9253‒1.087) | 0.9448 |
| Multiple sclerosis | Mixed conductive and sensorineural hearing loss | 21 | Weighted median | 0.9795 (0.9301‒1.0315) | 0.4325 |
| Multiple sclerosis | Mixed conductive and sensorineural hearing loss | 21 | Weighted mode | 0.9923 (0.9354‒1.0527) | 0.8007 |
| Multiple sclerosis | Sensorineural hearing loss | 20 | Inverse variance weighted | 1.0158 (0.997‒1.0349) | 0.0992 |
| Multiple sclerosis | Sensorineural hearing loss | 20 | MR Egger | 1.0254 (0.9926‒1.0593) | 0.1485 |
| Multiple sclerosis | Sensorineural hearing loss | 20 | Weighted median | 1.0209 (1.0014‒1.0408) | 0.0356 |
| Multiple sclerosis | Sensorineural hearing loss | 20 | Weighted mode | 1.0217 (0.9998‒1.044) | 0.0669 |
| Ankylosing spondylitis | Sensorineural hearing loss | 22 | Inverse variance weighted | 1.1722 (1.0941‒1.2558) | 0 |
| Ankylosing spondylitis | Sensorineural hearing loss | 22 | MR Egger | 1.2694 (1.1314‒1.4242) | 6.00E-04 |
| Ankylosing spondylitis | Sensorineural hearing loss | 22 | Weighted median | 1.1957 (1.0916‒1.3097) | 1.00E-04 |
| Ankylosing spondylitis | Sensorineural hearing loss | 22 | Weighted mode | 1.2073 (1.1125‒1.3103) | 2.00E-04 |
| Rheumatoid arthritis | Mixed conductive and sensorineural hearing loss | 22 | Inverse variance weighted | 1.0463 (0.9422‒1.1619) | 0.3977 |
| Rheumatoid arthritis | Mixed conductive and sensorineural hearing loss | 22 | MR Egger | 1.0235 (0.8339‒1.2563) | 0.8263 |
| Rheumatoid arthritis | Mixed conductive and sensorineural hearing loss | 22 | Weighted median | 0.9563 (0.836‒1.0939) | 0.5145 |
| Rheumatoid arthritis | Mixed conductive and sensorineural hearing loss | 22 | Weighted mode | 0.9719 (0.8207‒1.1509) | 0.7441 |
| Rheumatoid arthritis | Sensorineural hearing loss | 21 | Inverse variance weighted | 0.9682 (0.9367‒1.0008) | 0.0555 |
| Rheumatoid arthritis | Sensorineural hearing loss | 21 | MR Egger | 0.9563 (0.8953‒1.0215) | 0.2003 |
| Rheumatoid arthritis | Sensorineural hearing loss | 21 | Weighted median | 0.9693 (0.9234‒1.0176) | 0.2086 |
| Rheumatoid arthritis | Sensorineural hearing loss | 21 | Weighted mode | 0.9705 (0.9229‒1.0205) | 0.256 |
| Systemic lupus erythematosus | Mixed conductive and sensorineural hearing loss | 14 | Inverse variance weighted | 0.9879 (0.9467‒1.0308) | 0.5745 |
| Systemic lupus erythematosus | Mixed conductive and sensorineural hearing loss | 14 | MR Egger | 1.0043 (0.9142‒1.1033) | 0.9296 |
| Systemic lupus erythematosus | Mixed conductive and sensorineural hearing loss | 14 | Weighted median | 0.9971 (0.9412‒1.0562) | 0.9207 |
| Systemic lupus erythematosus | Mixed conductive and sensorineural hearing loss | 14 | Weighted mode | 0.9379 (0.8522‒1.0323) | 0.2131 |
| Systemic lupus erythematosus | Sensorineural hearing loss | 13 | Inverse variance weighted | 0.9953 (0.9795‒1.0114) | 0.5673 |
| Systemic lupus erythematosus | Sensorineural hearing loss | 13 | MR Egger | 0.9869 (0.954‒1.0209) | 0.4603 |
| Systemic lupus erythematosus | Sensorineural hearing loss | 13 | Weighted median | 0.9973 (0.9764‒1.0186) | 0.7997 |
| Systemic lupus erythematosus | Sensorineural hearing loss | 13 | Weighted mode | 0.9958 (0.9657‒1.0268) | 0.7911 |
| Ulcerative colitis | Sensorineural hearing loss | 17 | Inverse variance weighted | 1.0197 (0.9909‒1.0493) | 0.1826 |
| Ulcerative colitis | Sensorineural hearing loss | 17 | MR Egger | 1.1101 (1.0031‒1.2286) | 0.0617 |
| Ulcerative colitis | Sensorineural hearing loss | 17 | Weighted median | 1.0281 (0.9877‒1.0702) | 0.1748 |
| Ulcerative colitis | Sensorineural hearing loss | 17 | Weighted mode | 1.0206 (0.9506‒1.0957) | 0.582 |

**Table S9** Heterogeneity and pleiotropy post-outlier removal.

| **Exposure** | **Outcome** | **Heterogeneity** | | **Pleiotropy** | |
| --- | --- | --- | --- | --- | --- |
|  |  | **Q statistic (IVW)** | **p-value** | **MR-Egger Intercept** | **p-value** |
| Multiple sclerosis | Mixed conductive and sensorineural hearing loss | 28.43594 | 0.099475 | -0.00412 | 0.736682 |
| Multiple sclerosis | Sensorineural hearing loss | 35.36663 | 0.012612 | -0.00342 | 0.49577 |
| Ankylosing spondylitis | Sensorineural hearing loss | 21.4884 | 0.429486 | -0.00535 | 0.107791 |
| Rheumatoid arthritis | Mixed conductive and sensorineural hearing loss | 31.75087 | 0.062037 | 0.003654 | 0.807672 |
| Rheumatoid arthritis | Sensorineural hearing loss | 16.20407 | 0.703886 | 0.001883 | 0.677007 |
| Systemic lupus erythematosus | Mixed conductive and sensorineural hearing loss | 7.061365 | 0.898962 | -0.00726 | 0.70614 |
| Systemic lupus erythematosus | Sensorineural hearing loss | 7.601398 | 0.815453 | 0.003767 | 0.585934 |
| Ulcerative colitis | Sensorineural hearing loss | 14.93915 | 0.529099 | -0.01353 | 0.107374 |

**Table S10** MR-PRESSO results post-outlier removal.

| **Exposure** | **Outcome** | **Raw** | | **Outlier corrected** | | **Global p** | **Number of outliers** | **Distortion p** |
| --- | --- | --- | --- | --- | --- | --- | --- | --- |
|  |  | **OR (95% CI)** | **p** | **OR (95% CI)** | **p** |  |  |  |
| Multiple sclerosis | Mixed conductive and sensorineural hearing loss | 0.9914 (0.9477‒1.0371) | 0.711825 | NA (NA‒NA) | NA | 0.1292 | NA | NA |
| Multiple sclerosis | Sensorineural hearing loss | 1.0158 (0.997‒1.0349) | 0.115667 | NA (NA‒NA) | NA | 0.0225 | NA | NA |
| Ankylosing spondylitis | Sensorineural hearing loss | 1.1722 (1.0941‒1.2558) | 0.000188 | NA (NA‒NA) | NA | 0.494 | NA | NA |
| Rheumatoid arthritis | Mixed conductive and sensorineural hearing loss | 1.0573 (0.9552‒1.1702) | 0.293919 | NA (NA‒NA) | NA | 0.0643 | NA | NA |
| Rheumatoid arthritis | Sensorineural hearing loss | 0.9775 (0.9462‒1.0099) | 0.185432 | NA (NA‒NA) | NA | 0.4262 | NA | NA |
| Systemic lupus erythematosus | Mixed conductive and sensorineural hearing loss | 0.9879 (0.9574‒1.0194) | 0.459748 | NA (NA‒NA) | NA | 0.8933 | NA | NA |
| Systemic lupus erythematosus | Sensorineural hearing loss | 0.9953 (0.9827‒1.0081) | 0.486091 | NA (NA‒NA) | NA | 0.834 | NA | NA |
| Ulcerative colitis | Sensorineural hearing loss | 1.0171 (0.9901‒1.0448) | 0.232842 | NA (NA‒NA) | NA | 0.5271 | NA | NA |

**Figure S1 Forest plots demonstrate genetic associations between AS and HL.** (A) AS association with CHL (unspecified); (B) AS association with MHL; (C) AS association with SNHL; (D) AS association with SIHL. Forest plots summarize the genetic associations between ADs and HL, with Odds Ratios (ORs) and 95% Confidence Intervals (95% CIs) for each SNP and the overall effect sizes derived from MR analyses.


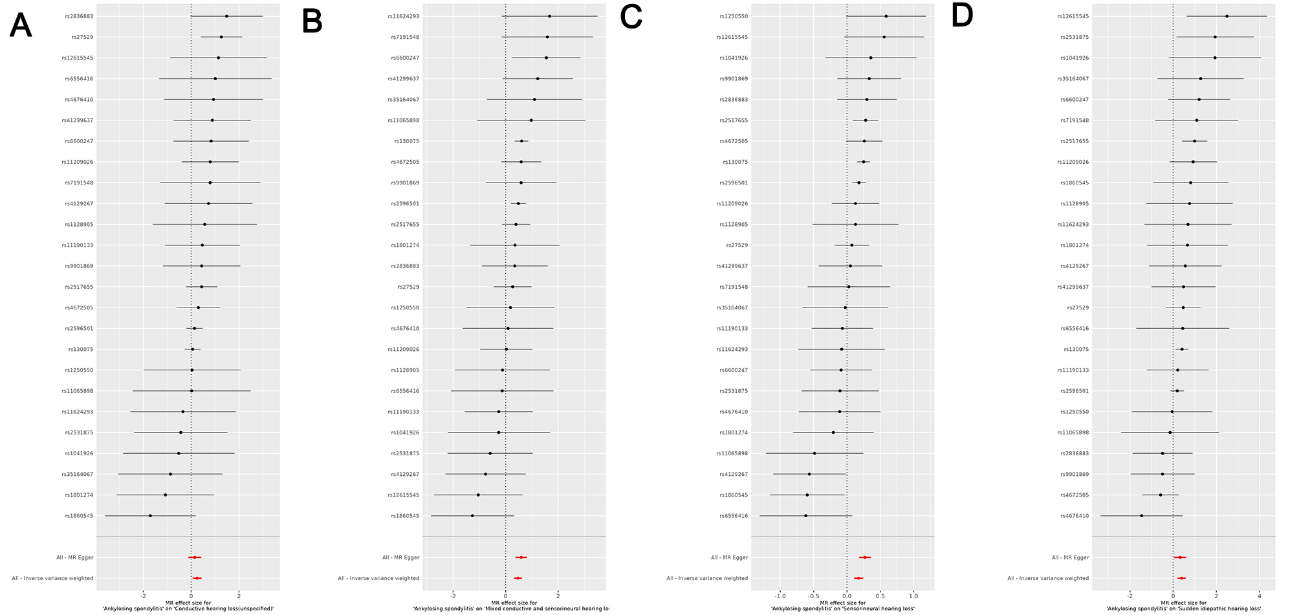


**Figure S2 Forest plots demonstrate genetic associations between CD and CHL (A), CD and SIHL (B), MS and SIHL (C), SLE and CHL (D), UC and MHL (E).** Forest plots summarize the genetic associations between ADs and HL, with Odds Ratios (ORs) and 95% Confidence Intervals (95% CIs) for each SNP and the overall effect sizes derived from MR analyses.


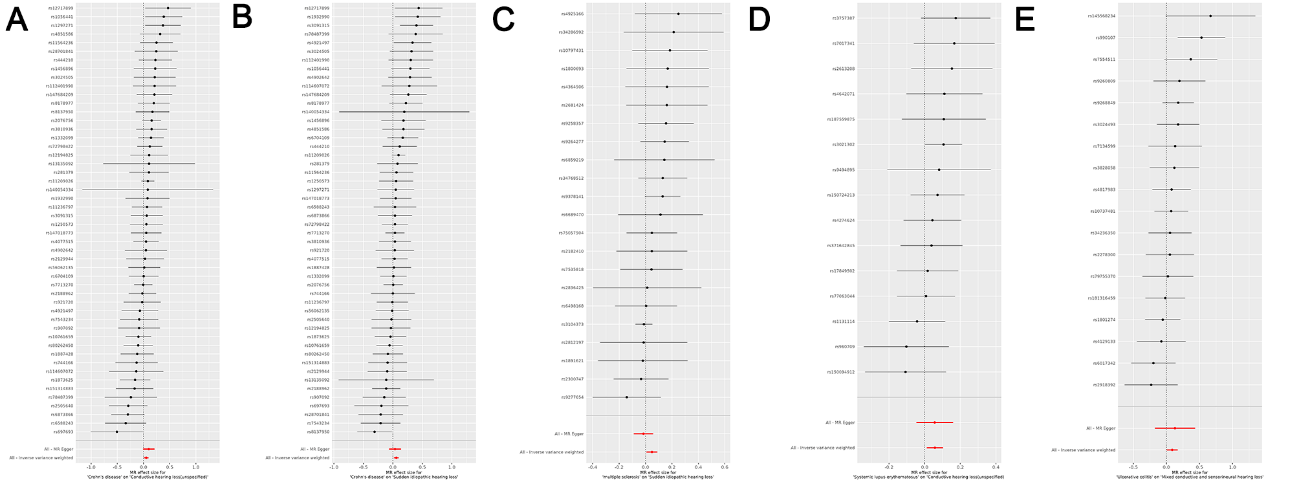


**Figure S3 Funnel plots assess horizontal pleiotropy for the association between AS and HL.** (A) AS association with CHL (unspecified); (B) AS association with MHL; (C) AS association with SNHL; (D) AS association with SIHL.


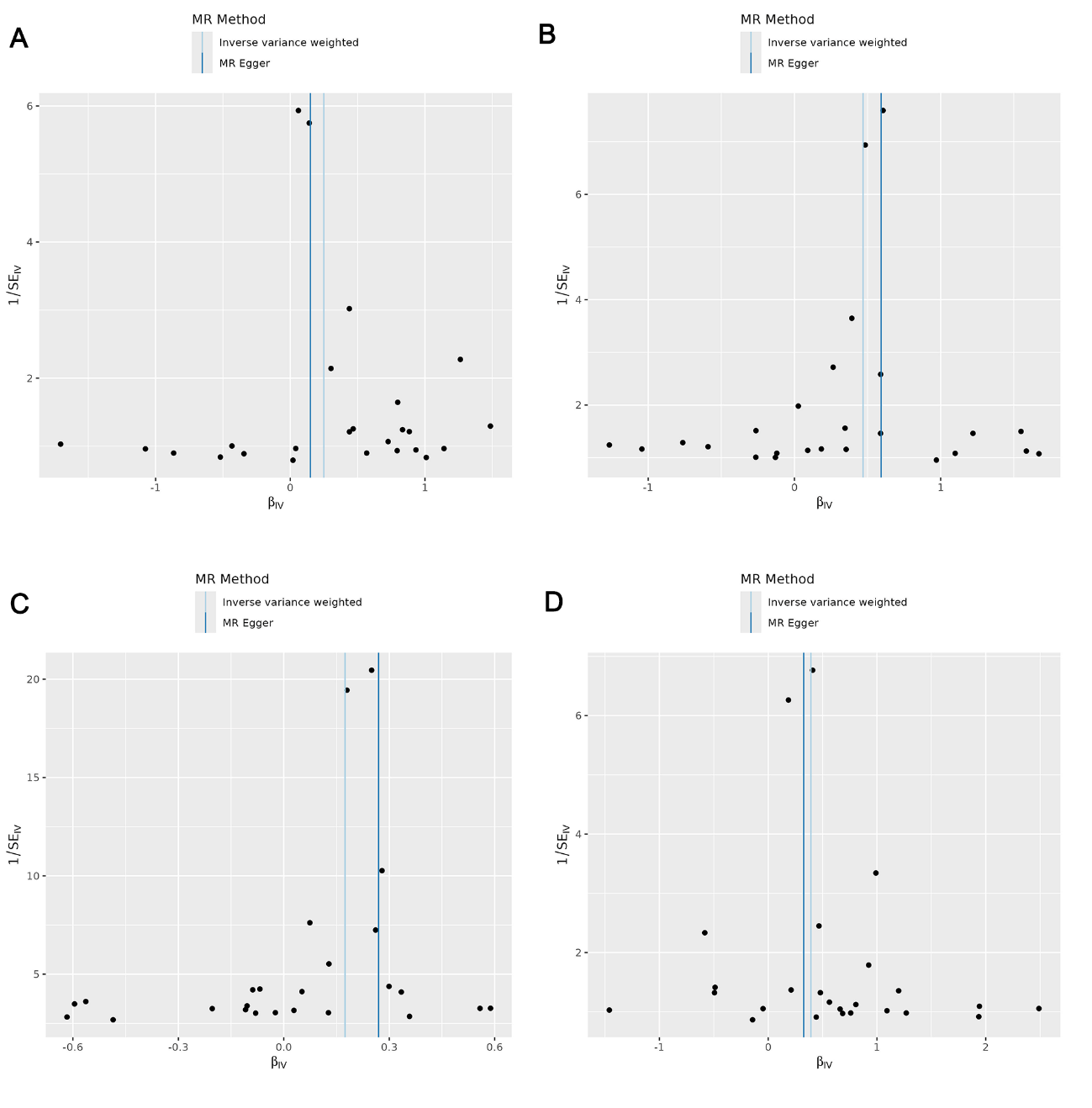


**Figure S4 Funnel plots assess horizontal pleiotropy for the association between CD and CHL (A), CD and SIHL (B), MS and SIHL (C), SLE and CHL (D), UC and MHL (E).**


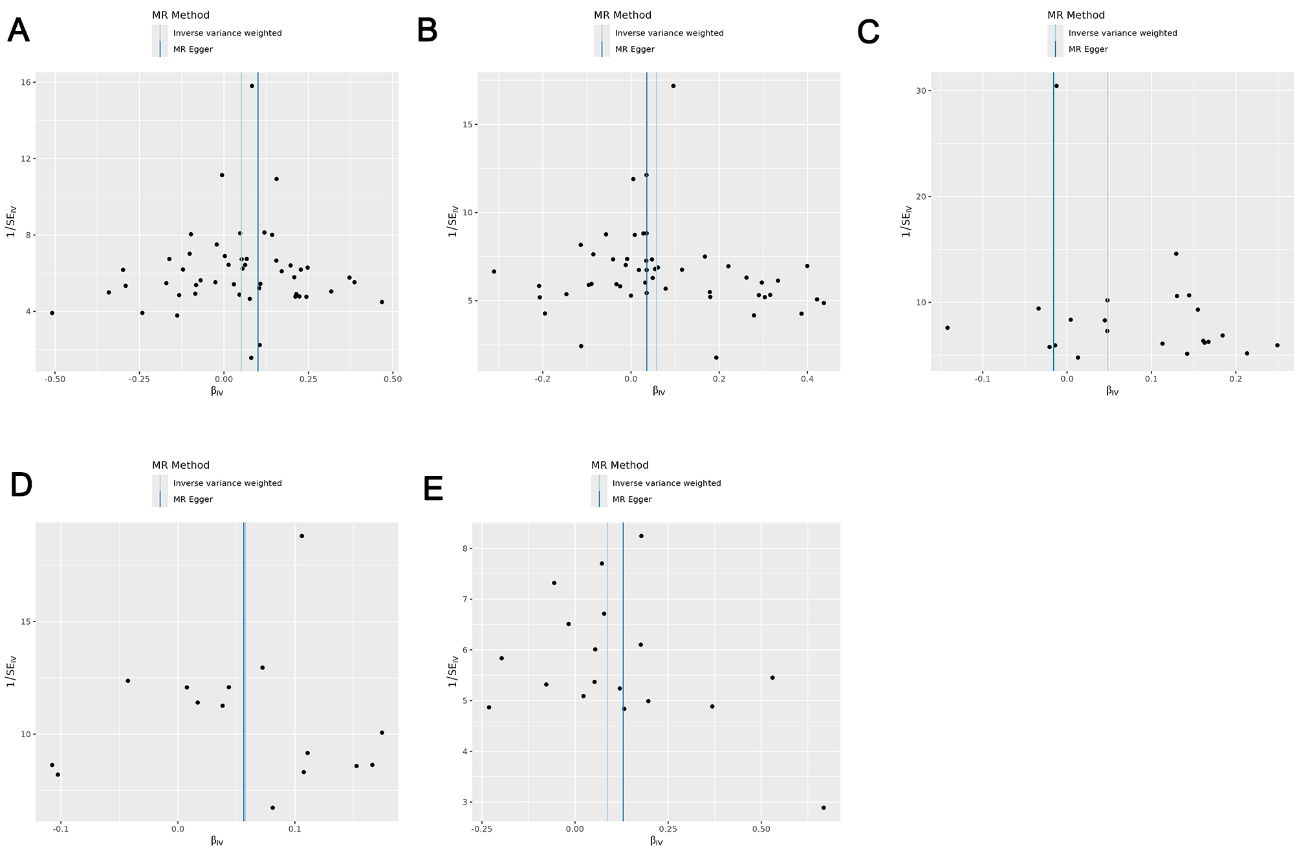


**Figure S5 Leave-one-out sensitivity analysis for the association between AS and HL.** (A) AS association with CHL (unspecified); (B) AS association with MHL; (C) AS association with SNHL; (D) AS association with SIHL.


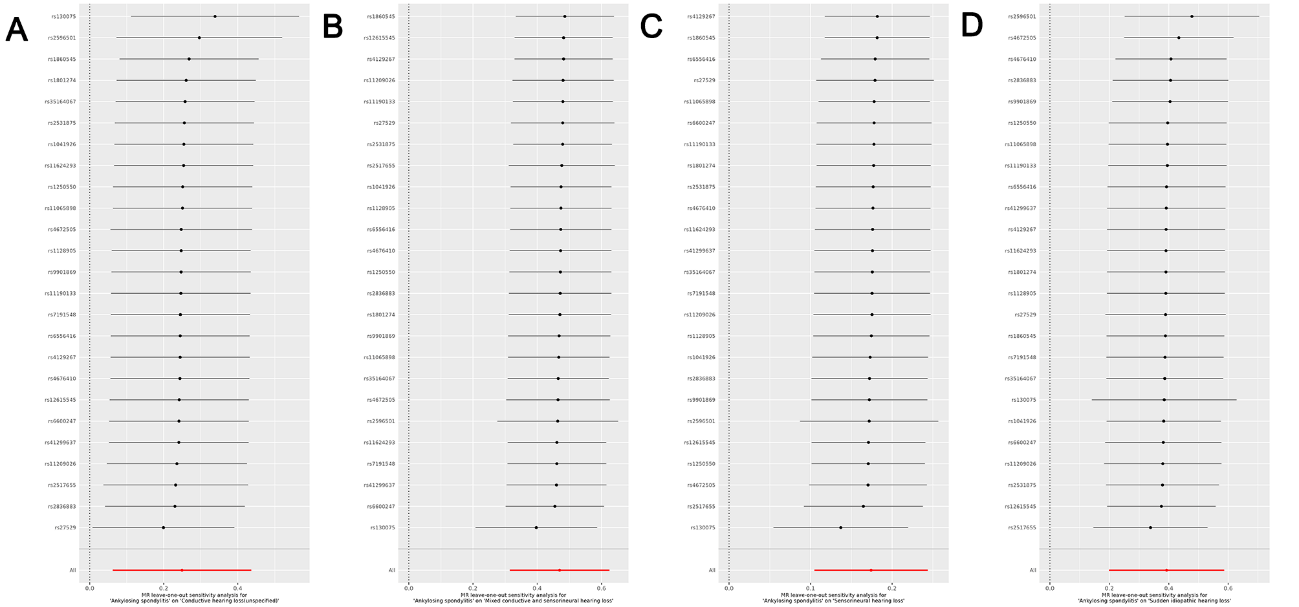


**Figure S6 Leave-one-out sensitivity analysis for the association between CD and CHL (A), CD and SIHL (B), MS and SIHL (C), SLE and CHL (D), UC and MHL (E).**


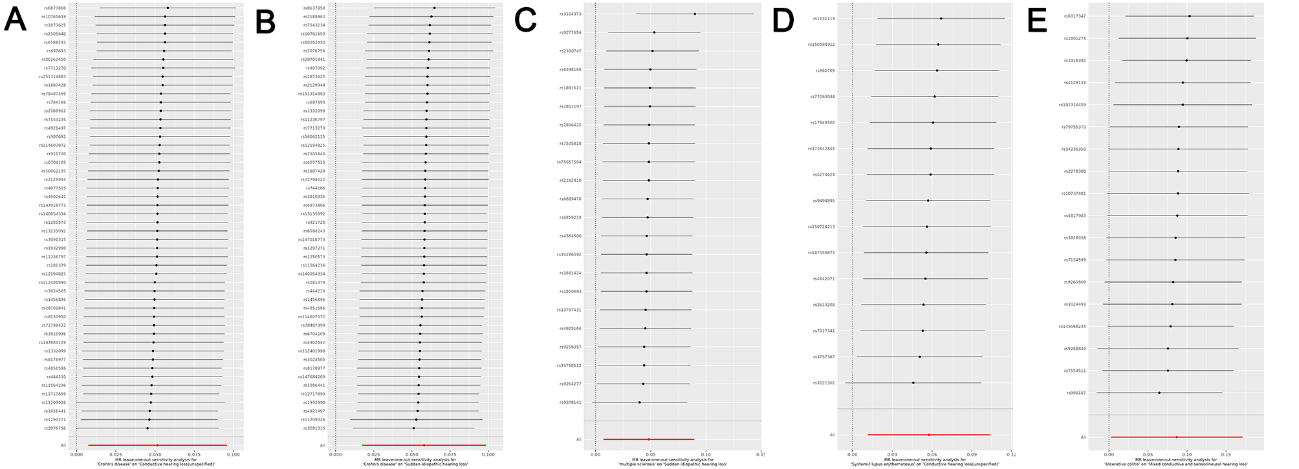

Supplement: Supplementary file 1 [file mmc1.docx]
